# Shift work and its effects on the cardiovascular system

**Published:** 2008

**Authors:** Thabo Mosendane, Tshinakaho Mosendane, Frederick J Raal

**Affiliations:** Reproductive Health and HIV Research Unit, Johannesburg; Reproductive Health and HIV Research Unit, Johannesburg; Endocrinology Unit, Department of Medicine, Johannesburg Hospital, Johannesburg

## Abstract

The practice of shift-work scheduling has long been part of normal work duties in emergency services such as health and security. It is only recently, in the wake of growing job opportunities and booming industries, where more employees are needed to keep services running over 24-hour periods that studies on the effects of shift work on workers’ health have begun to delve deeper.

The desynchronisation that occurs in circadian rhythms, with respect to sleep cycles, predisposes employees to coronary heart disease, gastrointestinal disturbances, increased risk of breast cancer and poor pregnancy outcomes. This literature review focuses on circadian rhythms, their molecular components, disturbances of these rhythms as a result of shift work and the adverse effects thereof on the cardiovascular system.

## Summary

There is no specific definition for the term shift work, but it is understood to be any work that is done outside of normal daytime working hours. Shift work has become an important part of many industries worldwide and is now considered a norm for some, with approximately 22% of the population in industrialised countries performing some type of shift work.^1^,^2^

Shift systems vary with respect to their structure, and in particular: the presence/absence of night work; the duration of the duty period (from six to 12 hours); the number of workers who cover the whole working time (two, three, four or more shifts); the interruption of the weekend or work done on a Sunday (continuous/discontinuous); if workers stay on a given shift, or alternate between the different shifts (permanent/rotating); the speed (fast/slow) and direction (clockwise/counterclockwise) of the shift rotation; the start and finish times of the duty periods; and the regularity/irregularity and length of the shift cycle.3 For the purpose of this article, the term shift work is used to encompass all work done between 16:00 and 07:00, or over a 24-hour period, unless stated otherwise.^3^

Approximately 15% of healthy individuals do not adapt adequately to the effects of shift work, therefore the performance of regular night duties is associated with a relatively high health risk.^4^ In his thesis *Long shifts, short rests and vulnerability to shift work*, John Axelsson explains that there is a large individual variation in tolerance to shift work, but it is not yet clearly understood why some individuals are more tolerant than others.^5^ No shift system has yet been found to be the most advantageous, but it seems that if a worker is able to choose his/her own hours in a shift-work system, it improves that individual’s ability to adapt to shift work.^6^

The effects of shift work can manifest in various forms of illness including peptic ulcer disease,^7^ breast cancer^8^ and obstructive sleep apnea.^9^ Hypertension, left ventricular hypertrophy, coronary heart disease and myocardial infarction are found more frequently and tend to be more severe in night-shift workers than in day workers.^2^ These problems are the result of the conflict that occurs between displaced work hours and the output of the biological clock.^10^

This review will focus on circadian rhythms, their molecular components and the effects of shift work on the cardiovascular system.

## Shift work and the cardiovascular system

The cardiovascular system in all mammals is highly organised with regard to time.^11^ Epidemiological studies have clearly documented that cardiovascular-related events, such as myocardial infarction, stroke and arrhythmias have the highest incidence of morbidity and mortality in the early hours of the morning, as opposed to occurring randomly.^12^

One of the first reviews to suggest an association between shift work and heart disease was published in 1984.^13^ Since then, there have been numerous studies on the topic, all concluding that there is a strong association between shift work and cardiovascular disease.^14^ In a review of 17 studies that were done on shift work and cardiovascular disease,^15^ it was calculated that shift workers had a 40% increased risk of cardiovascular disease compared with day workers.^16^ There is also evidence showing that individuals who have performed over six years of shift-work duties are at higher risk of developing cardiovascular disease.^17^

The exact mechanisms by which shift work causes cardiovascular disease are still not completely understood, but it is thought that the main contributing factors include disturbed circadian rhythms, and confounding factors such as smoking, poor eating habits, and social problems causing stress,^18^,^19^ which are common among shift workers.

## Circadian rhythms

These rhythms are controlled by intracellular molecular clocks that allow the organism to prepare itself for an anticipated stimulus. 20 Circadian rhythms are found in all species, in virtually all organs, as well as in tissues.^21^ The fungus *Neurospora crassa* makes asexual spores every 22 hours in constant darkness.^22^ In plants (*Arabidopsis thaliana* being the plant genetic model), numerous circadian rhythms exist and regulate functions such as leaf and petal movement, growth rates, opening and closing of stomatal pores, discharge of fragrances and the expression of many aspects of photosynthesis.^23^ The fruit fly *Drosophila melanogaster* shows a clear circadian locomotor rhythm in light cycles and constant darkness.^24^ In animals, more than 100 circadian rhythms have been identified, each with its own rhythm, which is able to influence various functions such as body temperature, blood pressure, heart rate and hormonal levels.

Two groups of circadian clocks have been described in mammals, namely central circadian clocks and peripheral circadian clocks.^33^ The former is located in the suprachiasmatic nucleus (SCN), above the optic chiasm in the hypothalamus. Here, it generates 24-hour endogenous circadian rhythms that allow for the coordination of physiological, metabolic and behavioural activities with external light/dark cycles, and anticipates daily environmental changes.^25^ Peripheral clocks are found in all tissues, including tissues within the central nervous system, in non-SCN cells.^26^,^27^ It is still unclear how these peripheral clocks are synchronised by the central SCN clock, although the involvement of neurohumoral stimuli is crucial.^28^

A significant characteristic of circadian rhythms is their ability to be synchronised (or entrained) by zeitgebers (external time cues).^29^ Light is the most potent stimulus for synchronising endogenous rhythms. Synchronisation is dependent upon the timing, intensity, duration and the wavelength of light.^30^ Photic information in mammals is transmitted via the retinothalamic tract to the SCN.^31^ Non-photic stimuli such as scheduled voluntary exercise, food, exogenous melatonin or serotonergic activation are also capable of shifting the endogenous circadian rhythms.

Nocturnal feeding has been shown to act as an entrainer on circadian rhythms, and affects the desynchronisation of these rhythms.^32^ An abrupt change in the feeding time schedule (from day to night) for several days gradually shifts the phases of the peripheral clocks, but not the central clock.^33^

Exogenous melatonin has acute sleepiness-inducing and temperature-lowering effects during the biological daytime, and when suitably timed, it will shift the phase of the human circadian clock to earlier (advance phase shift) or later (delay phase shift) times.^34^

After an abrupt change in an individual’s habits and/or environment, resynchronisation of rhythms can be achieved by a brief nap during the day or a transient nocturnal awakening that does not cause a change in the body’s endogenous clock.^32^

When the change occurs over a prolonged period (approximately five days), a mismatch between the endogenous and exogenous components of the rhythm occurs.^35^ It is this desynchronisation that is associated with poor health. Short-term adverse effects of such desynchronisation include sleep disturbances, shift-lag syndrome, increased risk of errors and workrelated accidents. The long-term effects result in disturbances of the cardiovascular and gastrointestinal systems,^36^ impaired glucose and lipid metabolism, reproductive difficulties and breast cancer.^37^

## Molecular components of mammalian circadian rhythm

The central clock inherently runs over a period of 25 hours, but is synchronised to 24 hours by zeitgebers,^38^ where light is the predominant signal for the synchronisation of circadian clocks.^39^ Light signals are carried to the SCN via light-sensitive ganglion cell photoreceptors that contain melanopsin, an opsin-based photo-pigment that is sensitive to blue light.^5^

To generate circadian signals, the SCN clock relies on feedback loops. The positive components of mammalian circadian clocks are two basic helix-loop-helix PAS-containing transcription factors, namely CLOCK (circadian locomotor output cycles kaput) and BMAL1 (brain and muscle arylhydrocarbon receptor nuclear translocator-like protein 1).^40^ These transcription factors form heterodimers and activate the transcription of three period genes (*per1*, *per2* and *per3*) and two cryptochrome genes (*cry1* and *cry2*)^41^ by binding to the E-box elements within promoter regions in various target genes.^42^

**Fig. 1.**
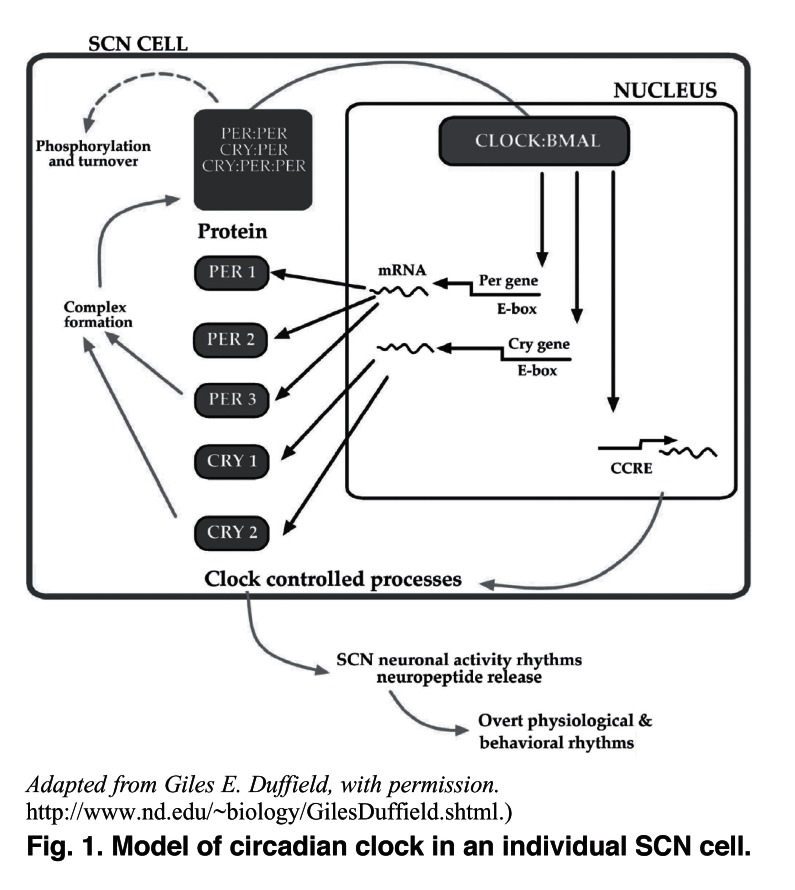
Model of circadian clock in an individual SCN cell.

The PER protein accumulates in the cytoplasm and translocates into the nucleus to form a complex with the CRY protein.^43^ The complex formed impairs phosphorylation and inhibits the CLOCK/BMAL1-dependent transcriptional activation, resulting in a decrease in the transcription of *per* and *cry* genes.^33^ This constitutes the core negative component of the feedback loop (Fig. 1).

Some nuclear orphan receptor genes, such as *rev-erb-α, rev-reb-β, ror-α and ror-β* are activated by CLOCK/BMAL1 heterodimers and produce proteins that differentially modulate BMAL1 transcription.^44^ *Rev-erb-α* suppresses BMAL1 gene transcription by binding to the retinoic acid-related orphan receptor-response elements (RORE) in the BMAL1 promoter region and *ror-α* activates its transcription.^45^ Together, *rev-erb-α* and *ror-α* suppress BMAL1 mRNA levels during the day and early night, but as *rev-erb-α* declines at night, BMAL1 is activated.^44^

Studies show that *Bmal1*^–/–^ mice not only lose the regularity of circadian oscillation, but also exhibit a variety of other phenotypes including decreased locomotor activity, reduced body weight, progressive joint disease and shortened life span.^46^ The gene encoding BMAL1 maps to human chromosome 11p15.2. In rats, it is located in a region of chromosome 1q34, harbouring the quantitative trait loci for blood pressure, type 2 diabetes mellitus, body weight, cardiac mass and kidney mass.^47^

## Disturbed circadian rhythms and the cardiovascular system

The expression of some genes in the heart oscillates with circadian rhythmicity (the heart expresses all forms of the *cry* and *per genes*).^48^ In pathological conditions, rhythmic expression of CLOCK genes in the heart and blood vessels and their output genes are altered. This alteration may disturb the ability of the heart to adapt to external stimuli and may accelerate tissue damage.^49^

Animal studies have recently identified BMAL2, a CLOCK in the vascular endothelial cells. BMAL2 forms a complex with CLOCK, and binds to the E-box elements upstream of the *pai*-1 gene to activate the PAI-1 promoter and induce PAI-1 mRNA expression.^50^ Thus BMAL2 regulates the circadian oscillation of PAI-1 gene expression in endothelial cells.^50^ Increased expression of PAI-1 activity is associated with an increased risk of acute myocardial infarction.^51^ PAI-1 promoter activity is inhibited by fundamental components of the central clock, including *per1*, 2 and *cry1*, 2.^49^

Thrombomodulin (TM), an essential co-factor for protein C activation, is also regulated by peripheral circadian clocks in the vascular endothelium. Circadian oscillation of TM gene expression may also contribute to the circadian variation of cardiovascular events.^52^

Furthermore, homozygous *Clock* mutant mice have impaired feeding rhythms, become hyperphagic and obese, and develop the metabolic syndrome.^53^ CLOCK also plays an important role in lipid metabolism by regulating the transcription of peroxisome proliferator-activated receptor α (PPARα) in mice.^54^ PPARα is a member of the nuclear receptor super-family and is involved in the activation of numerous pathways of lipid metabolism, including fatty acid uptake, beta-oxidation, transport into peroxisomes and omega-oxidation of unsaturated fatty acids.^55^

## Shift work and cardiovascular disease

The links between shift work and the increase in metabolic risk factors for cardiovascular disease (CVD) have been well documented.^56^ Desynchronisation of circadian clocks, which may occur as a result of shift work, leads to hypertension, dyslipidaemia, insulin resistance and obesity.^57^

## Hypertension

**Fig. 2.**
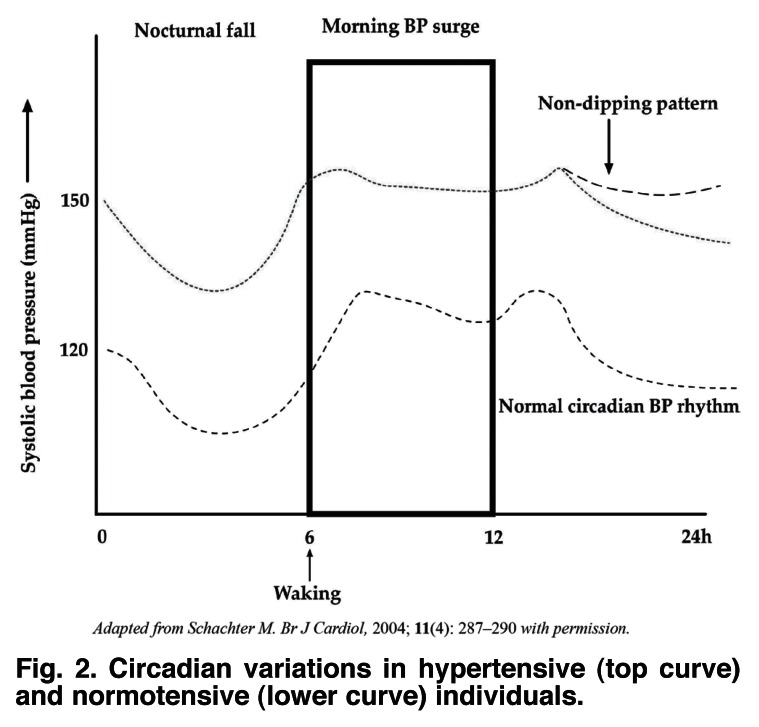
Circadian variations in hypertensive (top curve) and normotensive (lower curve) individuals.

Shift work changes the diurnal variation of blood pressure from a dipper to a non-dipper pattern,^58^ thus increasing the risk of hypertension among night-shift workers. The normal daily circadian blood pressure rhythm is characterised by a nocturnal fall and diurnal rise. Individuals who show a nocturnal blood pressure (BP) fall of at least 10% of mean arterial pressure (MAP) are classified as dippers.^59^ Non-dippers are characterised by a lack of, or a very limited nocturnal BP fall. Cardiovascular outcomes are worsened in individuals who have an excessive morning BP surge and in those who lack the normal nocturnal BP fall (Fig. 2).

Several studies have investigated the relationship between the lack of nocturnal BP fall (the non-dipping pattern) and cardiovascular risk and have shown it to be associated with an increase in target-organ damage (heart, brain, kidney), a greater frequency of cardiovascular events (stroke, myocardial infarction, etc), and higher cardiovascular mortality in both hypertensive and normotensive individuals.^60^-^62^

## Dyslipidaemia

Plasma lipid concentrations also exhibit circadian rhythmicity.^63^ Night-shift workers are reported to have a higher incidence of heart disease and also demonstrate higher triglyceride levels compared with matched day workers.^64^ The principal consequence of ingesting a meal at night (as is the case with many shift workers) is the production of higher concentrations of serum triacylglyerol levels and lower concentrations of cholesterol-rich lipoproteins than after a daytime meal.^65^

A cohort of 25 healthy adults was studied by Rivera-Coll *et al.* in 1990, to investigate the dyslipidaemia associated with shift work. Blood samples were collected from the participants at four-hour intervals over a 24-hour period. The levels of cholesterol at various times of the day were documented and it was found that the circadian variation (given as a percentage of the total variation) for the ratio between high-density lipoprotein cholesterol (HDL-C) and total cholesterol was 5.6%. The circadian variation for HDL-C was 30.5%, for total cholesterol 31.6%, for low-density lipoprotein cholesterol (LDL-C) 33.5% and for triglycerides 38.5%.^66^

Masoumeh Ghiasvand and colleagues conducted a study among railroad workers and demonstrated that total cholesterol and LDL-C levels of shift workers were significantly higher compared to day workers, and concluded that shift work is a risk factor for lipid profile disturbance.^67^

## Glucose intolerance and obesity

Glucose tolerance has a diurnal variation that is partly the result of the variable levels of cortisol concentrations throughout the day.^68^,^69^ Glucose tolerance has been shown to have a decreasing trend during the day in normal individuals,^70^ therefore the intake of meals during the night results in the higher incidence of obesity^71^ and weight gain^72^ that is often associated with shift work.

Katherine Parks investigated shift patterns (day shifts versus day−night rotational shifts) and their interaction with age and years of shift-work exposure, as predictors of body mass index (BMI). It was found that for day-shift workers, age predicted BMI, but for day−night shift workers, the major predictor of BMI was duration of exposure to day−night shift work.^73^

In a small-scale retrospective study, nurses reported weight gain once they started a shift-work routine.^74^ The weight gain appeared to be the result of changes in dietary habits and exercise patterns, but the study of thermogenic responses to food with respect to time of day^75^ showed that approximately 15.9% of the energy content of the morning meal was used, while only 10% of the energy content in the evening meal was used. Therefore, a greater percentage of the energy in food eaten during the day is released as heat rather than taken up by the body and stored.

Abdominal obesity, elevated triglyceride levels (> 1.7 mmol/l), low HDL-C levels (< 1.03 mmol/l in males and < 1.29 mmol/l in females) and impaired glucose tolerance appear to cluster together more often in night-shift workers than in day workers,^14^,^76^,^77^ predisposing night-shift workers to the development of the metabolic syndrome as defined by the International Diabetes Federation (IDF).

## Confounding factors

It is possible that the association between shift work and cardiovascular disease may be strongly influenced by changes in dietary habits, reduced physical activity, increased smoking, heavy drinking and disruptions in psychosocial factors. It has been shown that shift workers experience irregular appetites that may be associated with weight gain, or loss in rare cases, because of a combination of high-fat snacking, infrequent eating during the day, over-eating at night and a lack of exercise.^78^ In a Swedish prospective study of occupational stress and ischaemic heart disease, it was found that smoking among shift workers was significantly increased,^79^ contributing greatly to CVD.

Shift work disrupts the family life and restricts the social life of those who perform it. Lipovcan and colleagues conducted a study to assess the quality of life, satisfaction in life, happiness, and the demands of work in shift workers and non-shift workers, and found that shift work had a negative effect on social and domestic life.^80^

## Limitations of health research on shift work

The methodology of research on shift work has several limitations. 14 Confounding factors may include: no clear definition of shift work, lack of consideration of ‘selection in’ and ‘selection out’ biases, no clear duration of involvement in shift work, a past history or a family history of myocardial infarction, less consideration of social class and no consistent results.^81^

Many studies focus on data from a single occupational group and make comparisons between day workers and shift workers as well as different rotational shifts.^82^ By performing such comparisons one must assume that the job demands are the same across the different shift systems, but this is not always the case.

Many companies advocate pre-employment screening, so one would assume that performing studies among this occupational group would influence study findings. This is referred to as the healthy shift-work hire effect.^81^

Differences in social class may occur between shift workers and day workers. Unskilled workers are more often involved in shift work, but previous studies have not controlled for social class where an association between shift work and cardiovascular disease was found.^83^

## Conclusion

Shift work may be associated with an increased risk of CVD for several reasons. Disturbed circadian rhythms, lifestyle changes and psychosocial stress are all factors that are frequently mentioned.

All staff members performing shift-work duties should be offered regular occupational health services which should include the screening of risk factors for cardiovascular diseases, such as: a history of shift work, smoking, blood pressure, obesity, alcohol use, blood lipids, physical inactivity and work stress.

Although shift work has been regarded as a risk for cardiovascular disease for the last decade,^84^ limited data exist on the connection between shift work and ill health in South Africa. Ergonomic shift criteria have been introduced with regard to interventions in shift scheduling, which aim to optimise the well being of shift workers and identify ill health at an early stage.^85^
